# GaAs-based resonant tunneling diode (RTD) epitaxy on Si for highly sensitive strain gauge applications

**DOI:** 10.1186/1556-276X-8-218

**Published:** 2013-05-08

**Authors:** Jie Li, Hao Guo, Jun Liu, Jun Tang, Haiqiao Ni, Yunbo Shi, Chenyang Xue, Zhichuan Niu, Wendong Zhang, Mifeng Li, Ying Yu

**Affiliations:** 1 Key Laboratory of Instrumentation Science & Dynamic Measurement, North University of China, Ministry of Education, Shanxi, 030051, China; 2 Science and Technology on Electronic Test & Measurement Laboratory, North University of China, Taiyuan, Shanxi, 030051, China; 3 State Key Laboratory for Superlattices and Microstructures, Institute of Semiconductors, Chinese Academy of Sciences, Beijing, 100083, China

**Keywords:** RTD epitaxy on Si, Strain gauge, Highly sensitive, Piezoresistive coefficient

## Abstract

As a highly sensitive strain gauge element, GaAs-based resonant tunneling diode (RTD) has already been applied in microelectromechanical system (MEMS) sensors. Due to poor mechanical properties and high cost, GaAs-based material has been limited in applications as the substrate for MEMS. In this work, we present a method to fabricate the GaAs-based RTD on Si substrate. From the experimental results, it can be concluded that the piezoresistive coefficient achieved with this method reached 3.42 × 10^−9^ m^2^/N, which is about an order of magnitude higher than the Si-based semiconductor piezoresistors.

## Background

In recent years, resonant tunneling diode (RTD) has attracted growing interest on the applications of highly sensitive strain gauge. Wen et al. explained this phenomenon as the meso-piezoresistance effect, which is the resonant tunneling current of the RTD tuned by the external mechanical strain [1]. Our previous study has already proved that the strain gauge sensitivity of the GaAs-based RTD can be one to two orders of magnitude higher than the traditional Si-based piezoresistive sensing elements [2-4]. Combining with the microelectromechanical system (MEMS) fabrication process on GaAs substrate, RTD has been fabricated as the embedded mechanical sensing element for different MEMS sensors: accelerometers [5] and hydrophone [6].

Compared to Si, GaAs is quite fragile, a property which limited its applications in the field of MEMS sensors especially as mechanical structures. Meanwhile, GaAs is quite expensive in terms of the material and fabrication process. To further expand the application fields of the excellent performances of GaAs-based mechanical sensing element, it is quite necessary to combine the highly sensitive GaAs-based strain gauge elements with the Si substrate.

Due to lattice mismatch, GaAs is quite difficult to be fabricated on Si substrate [7]. Researchers have already worked for many years to combine the advantage of Si-based materials with other semiconductor materials for application in microelectronics and photonics, and different technologies have been reported: direct GaAs-on-Si epitaxy, GaAs-on-Si growth through Ge buffer layers, GaAs-on-SOI epitaxy, GaAs-on-STO-Si epitaxy, bonding, etc. [8-10].

From the literatures reported, it can be concluded that different methods on the GaAs-on-Si epitaxy have already been developed, but still there is no report on the GaAs-based strain gauge elements on Si which could be applied on the MEMS sensor. In our previous research, we have developed a method to optimize the GaAs-on-Si substrate, which has greatly reduced their residual stress and surface defect density [11]. In this work, based on the surface optimization technology that we developed, the RTD structure was then grown on the optimized substrate; combining Raman spectroscopy and *I*-*V* characterizations, the stress–strain coupling effect from the Si substrate to GaAs-based RTD was tested. Finally, the piezoresistive coefficient of the RTD was characterized. This method gives us a solution to optimize the epitaxy GaAs layers on the Si substrate, which also proved the possibility of our future process of integrating GaAs-based RTD on the Si substrate for MEMS sensor applications.

## Experimental

Commercially available GaAs-on-Si wafers were used as the initial substrates in this experiment, which were purchased from Spire Corp., Bedford, MA, USA. The GaAs layers were grown directly on 3-in. Si wafers (with N^+^ doping concentrations of 5 × 10^16^ cm^−2^ and 350 μm in thickness). GaAs epilayers with a thickness of 2 μm were grown on (100)-oriented Si with 4° misorientation toward the (111) Si substrate. The initial density of the lattice defect of the purchased GaAs/Si wafers was about 10^8^ cm^−2^.

The GaAs-based optimization superlattice layers and RTD heterostructures were fabricated by molecular beam epitaxy using Veeco Mod-GEN II, Plainview, NY, USA. InGaAs/GaAs strain superlattice was used as the buffer layer to optimize the defects and residual stress of the substrate, and then the RTD heterostructures were grown on top as the strain sensing element.

The surface topography and cross-section of the epilayers were characterized by transmission electron microscopy (FEI Tecnai G2 F20, Hillsboro, OR, USA) and scanning electron microscopy (KYKY-1000B, Beijing, China). The stress–strain coupling effect was characterized by residual stress using the Renishaw inVia Raman microscope system (Gloucestershire, UK; the laser line is 514.5 nm, and the excitation beam power is 5 mW). The luminescence characteristics of the quantum well were observed using Fourier transform infrared spectrometer (Nicolet FTIR760, Appleton, WI, USA) with a power of 1 W and a wavelength of 632.8 nm.

**Figure 1:**
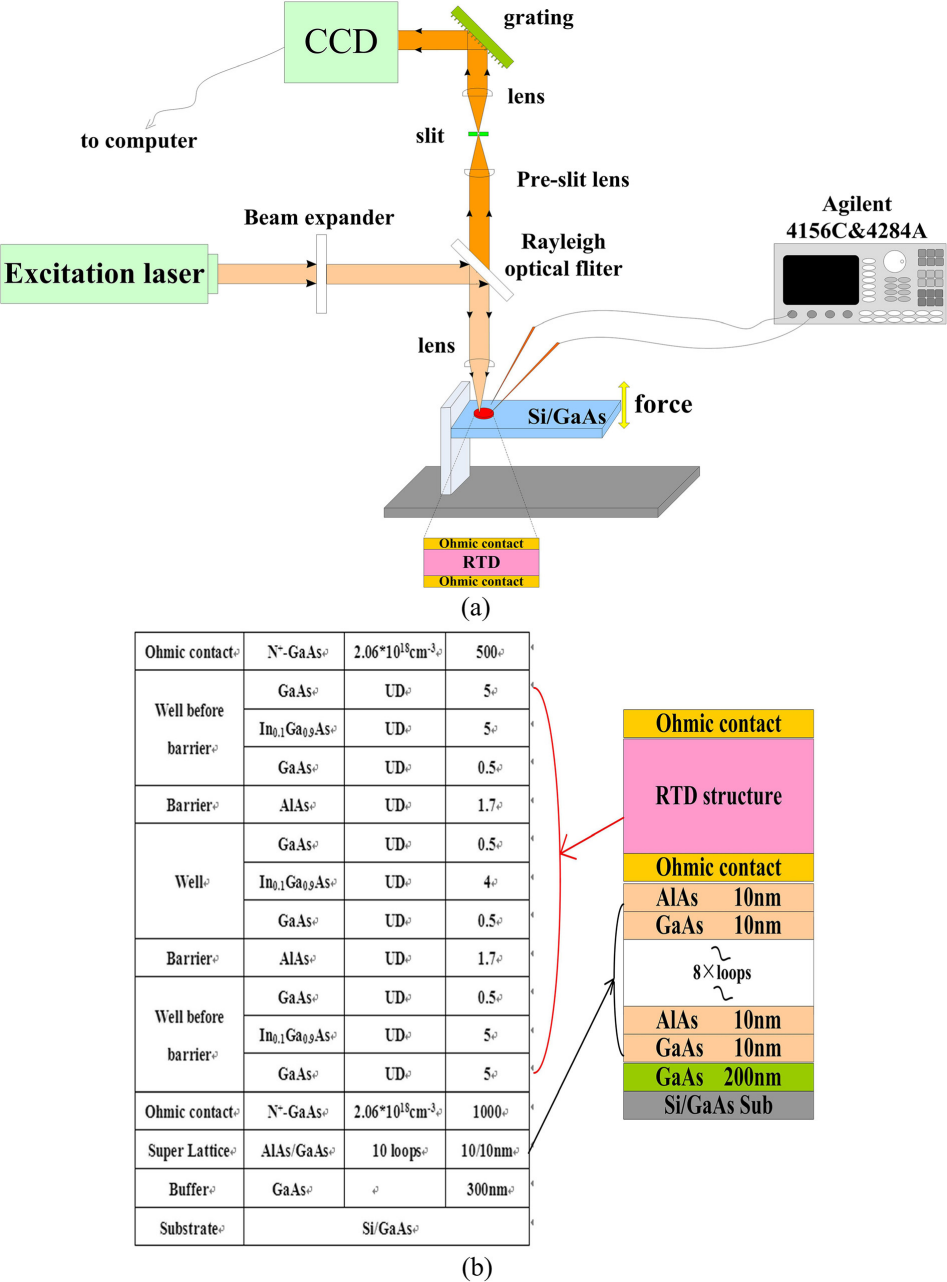
**Schematic diagram for testing.** (**a**) Schematic of the electrical and Raman characterization system, (**b**) the RTD with supperlattice structure.

The samples were cut into pieces of 0.5 cm × 2 cm for the stress–strain coupling effect test. The schematic of the setup used to strain the samples is provided in Figure 1. The sample was fixed on a homemade test setup from one end. The other end of the substrate was free to move. The micrometer was used to stress the sample from the free end. By tuning the micrometer, different stresses were applied. Another copper strain gauge element was pasted on the other side of the substrate, which was used to calibrate the applied stress quantitatively. The electrical responses were characterized by Agilent 4156C (Santa Clara, CA, USA).

## Results and discussion

**Figure 2:**
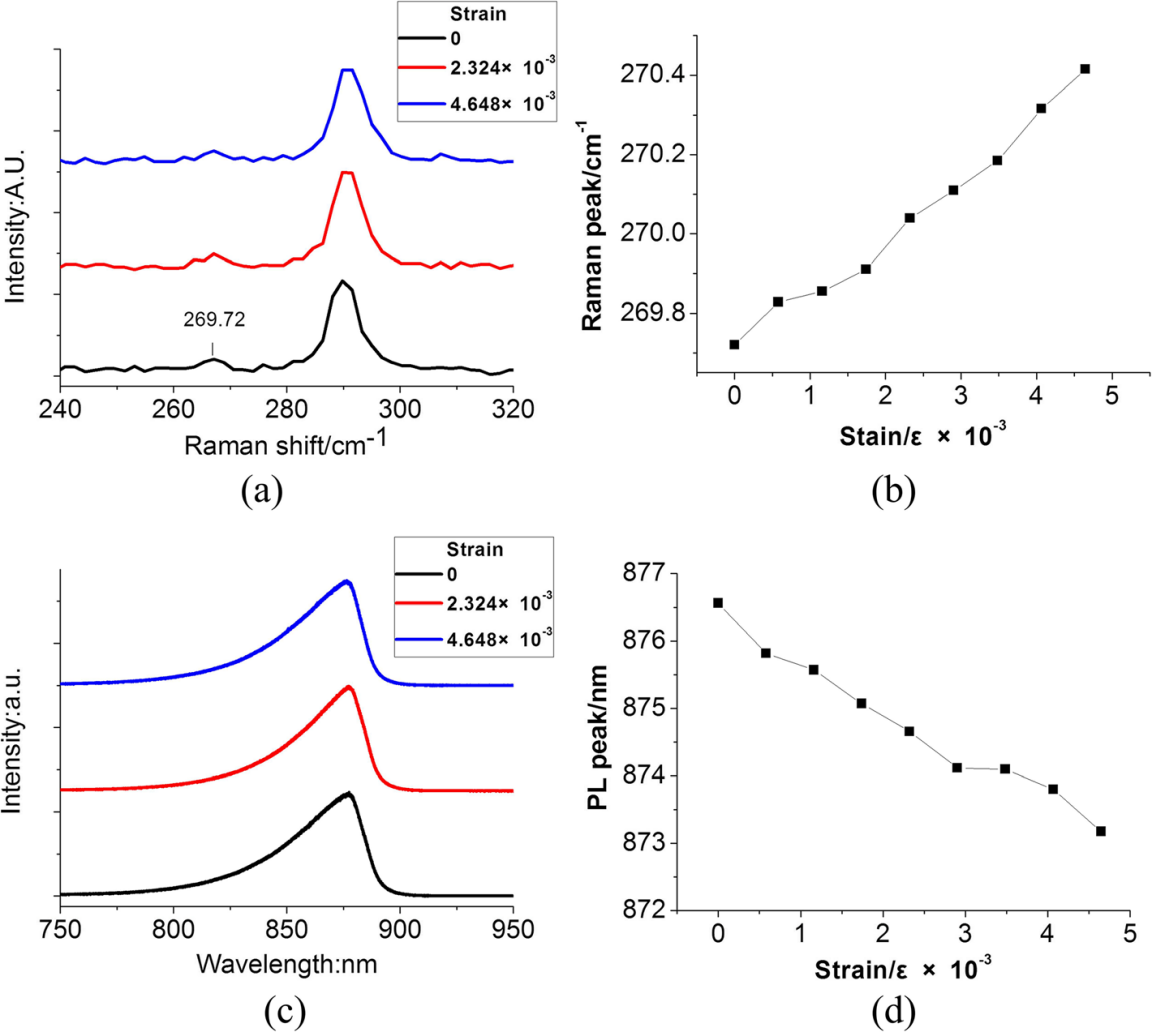
**Raman and PL characterizations of the GaAs-on-Si substrate.** (**a**) Raman spectrum of the substrate with and without strain, (**b**) Raman shift of GaAs under different strains, (**c**) the PL spectrum of the substrate with and without strain, and (**d**) the PL shift of GaAs under different strains.

The stress–strain coupling effect from the Si substrate to the GaAs layers was first characterized. The initial substrate was cut into samples of size 0.5 cm × 2 cm, with different strains applied on the samples. As shown in Figure 2a, without external strain, a Raman peak of 269.72 cm^−1^ was observed on the substrate, which has a Raman shift of 2.72 cm^−1^ with the intrinsic GaAs Raman peak. It means that there is residual stress on the sample surface from the calculation of the stress on GaAs [12]:

(1) $$\sigma_{\mathrm{GaAs}}=-576\Delta \omega =1.57\;\mathrm{GPa}.$$

As the stress on the substrate continues to increase, as shown in Figure 2b, the Raman peak was shifted from 269.72 to 270.415 cm^−1^, which means that there was a stress variation of 400.14 MPa. It can be explained by the fact that Raman scattering is related to the molecular rotation and range of transition between vibrational energies [13]. Raman spectroscopy can accurately measure the lattice vibration energy of materials. The lattice structure changes with stress, and the lattice vibration energy changes which leads to Raman peak shift.

The stress-induced strain in GaAs surface was also proved by the photoluminescence (PL) spectrum. As shown in Figure 2c, the substrate without any strain showed a PL peak in 876.56 nm, which has a blueshift of 6.56 nm with the intrinsic GaAs PL peak of 870 nm. We believe that this PL shift was caused by residual stress, which increased the bandgap of the GaAs. By increasing the stress, the PL peak was observed to further shift to 873 nm, as shown in Figure 2d.

**Figure 3:**
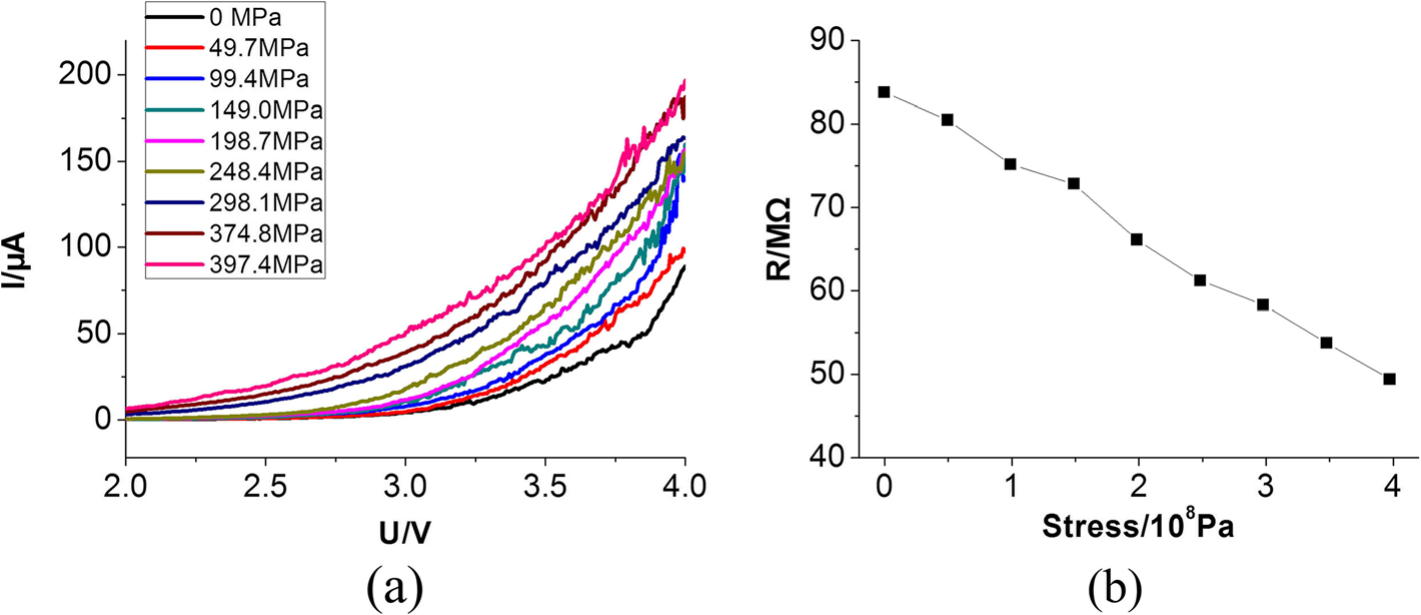
**Electrical characterizations of the GaAs-on-Si substrate.** (**a**) The *I*-*V* characteristics of wafer as a function of stress and (**b**) the resistance changes under different stresses.

The stress-resistance effect was then characterized. The *I*-*V* characteristics were measured with one electrode on the Si substrate and another electrode on the GaAs substrate. The *I*-*V* characterizations with different applied stresses are shown in Figure 3. From these test results, we have further calculated the piezoresistive coefficient of the GaAs on the Si substrate:

(2) $$\pi =\frac{\mathrm{\Delta R}}{\mathrm{R\tau}}=\frac{0.051184}{49.68\times 10^{-6}}=1.03\times 10^{-9},$$

where *π* is the piezoresistive coefficient and Δ*R* is the change in base resistance *R* in the function of stress *τ*.

This result is bigger than the Si-based semiconductor piezoresistors (*π* = 7.18 × 10^−10^ m^2^/N) [14,15].

The high piezoresistive coefficient of the GaAs on the Si substrate can be explained by the heterojunction structure between Si and GaAs. The piezoresistance effect of single-crystal Si can be attributed to the deformation of material structure, but GaAs-on-Si substrate consists of the deformation and carrier concentration in the built-in field of heterojunction structure. The resistance of the substrate can be calculated by the following [16]:

(3) $$R_{S}=\frac{1}{\mathrm{\sigma h}}=\frac{1}{\mathrm{eh}\left(\mu_{n}n+\mu_{p}p\right)}=\frac{1}{\mathrm{eh}\mu_{n}n},$$

where *σ* is the conductivity, *h* is the thickness, *e* is the electron charge, *n* and *p* are the carrier concentrations, and *μ*_*n*_ and *μ*_*p*_ are the mobilities. The heterojunction structure has increased the sensitivity of the strain gauge, which is one of the key reasons to use GaAs-based material as the strain gauge element.

Clear improvement of the piezoresistive coefficient of the GaAs on the Si substrate was concluded. There are still several problems which will hinder our future development of MEMS devices. First, the lattice defect has reached 10^8^ cm^−2^ which will greatly reduce the quality of the latter epitaxy layers. Second, the residual stress of the substrate reached 1.57 GPa, which will greatly reduce the sensitivity and reliability of the MEMS strain gauge sensing element.

We have also developed a method to optimize the GaA-on-Si substrate, which is based on an AlAs/GaAs matching superlattice structure. Using the matching superlattice, the density of lattice defect was calculated to be 1.41 × 10^6^ cm^−2^, which is about two orders of magnitude less than the initial defect density. Meanwhile, the residual stress in the optimized material is tensile stress, which is different from the stress in the wafer which is compressive stress. The value of residual stress reduces down to 232.13 MPa [11].

**Figure 4:**
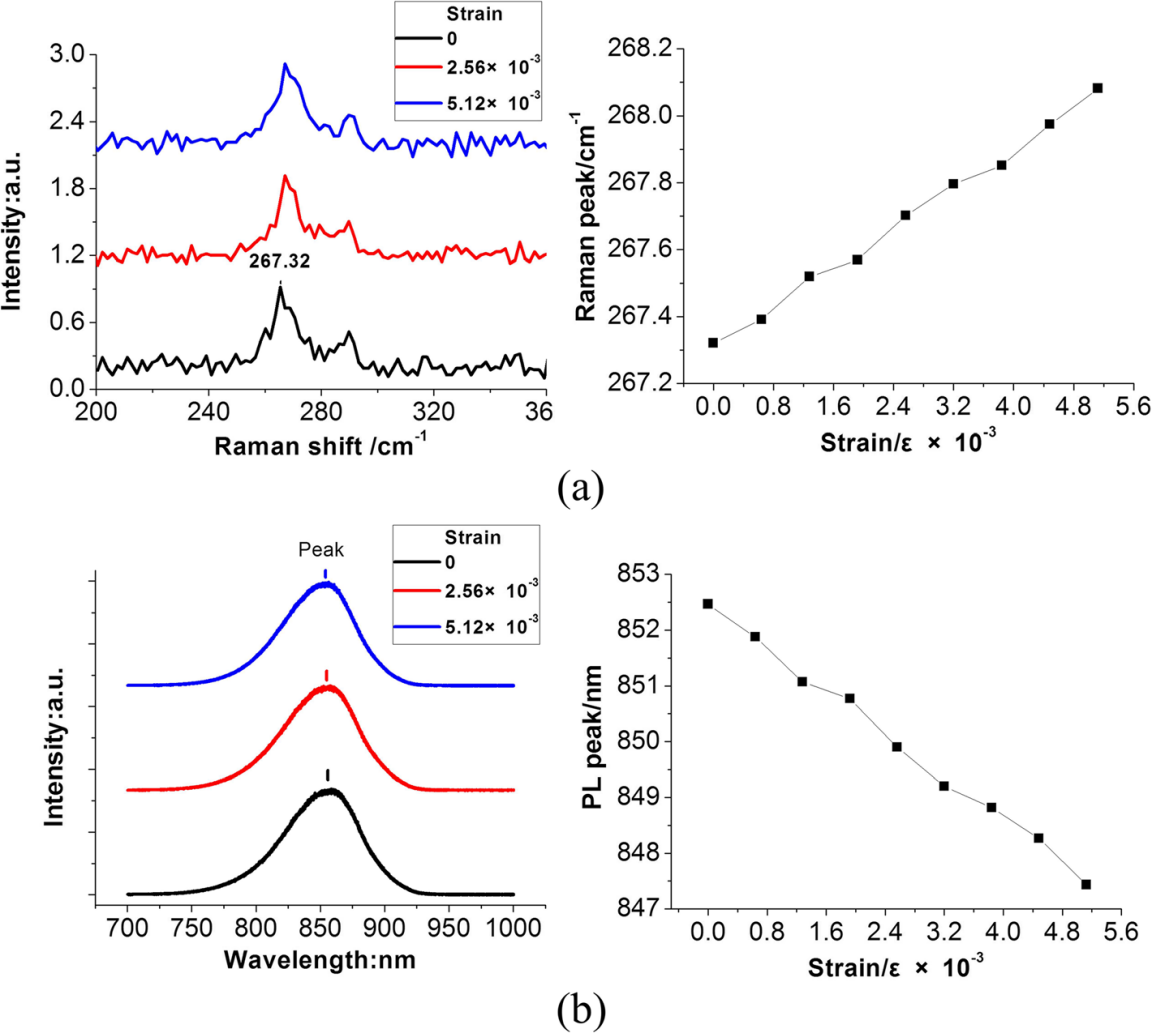
**Raman and PL characterizations of the RTD-on-Si substrate.** (**a**) The Raman spectrum and (**b**) PL spectrum of the sample under different strains.

The RTD supperlattice structure, as shown in Figure 1b, was then grown on the optimized GaAs-on-Si substrate. From the Raman spectrum shown in Figure 4a, it can be concluded that the longitudinal phonon spectroscopy becomes even stronger than the optimized substrate, which is more close to the standard Raman spectrum of GaAs crystal. It means that with the superlattice structure of RTD, the quality of the substrate material was further improved. This improvement was also proven by surface residual stress calculations. The peak of the Raman spectrum was shifted to 267.32 cm^−1^, which was 0.32 cm^−1^ shifted when compared with the optimized substrate. By calculating with Equation 1, the surface residual stress was reduced to 184.84 MPa, which is much smaller than the optimized substrate.

As shown in Figure 4a, the clear blueshift of the Raman spectrum was observed by external stress. With the stress increased from 0 to 5.13 × 10^−3^, the Raman peak was shifted from 267.32 to 268.08 cm^−1^, which means that a stress of 438.2 MPa was generated on the RTD. The same conclusion was obtained from the PL spectrum. In general, interatomic spacing becomes narrow with the stress. The smaller the distance between atoms, the larger the discrete energy level by the quantum energy level theory [17], which means that the bandgap becomes wider with the stress and the PL peak becomes small. As shown in Figure 4b, by increasing the stress, the peak shifted from 855.46 to 847.43 nm.

**Figure 5:**
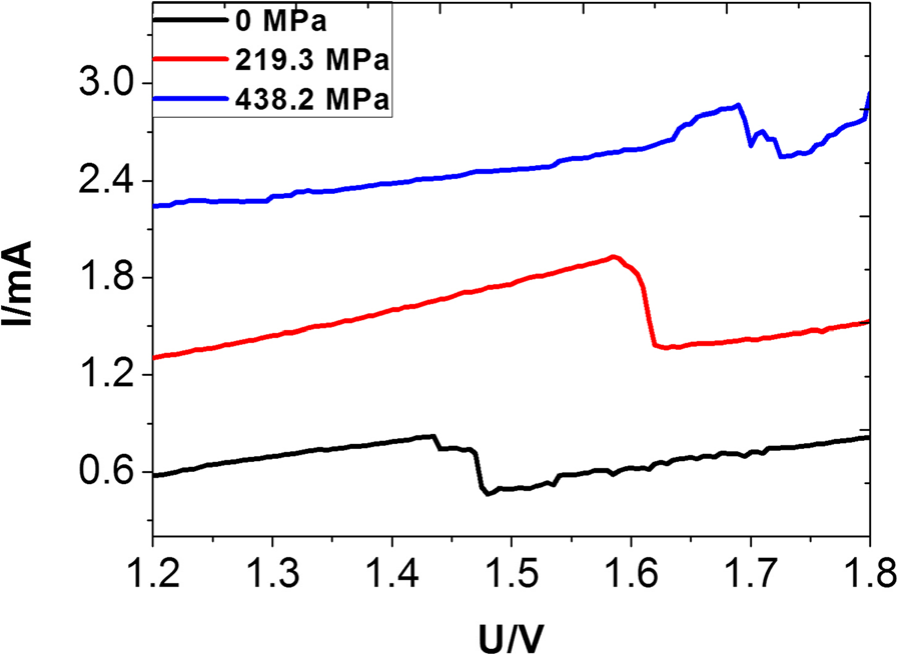
***I*-*V *characterizations of the RTD with different stresses.**

*I*-*V* characterizations of the RTD on the GaAs-on-Si substrate were done. The *I*-*V* characteristics of the GaAs-on-Si substrate and the RTD are shown in Figure 5. From the *I*-*V* characterizations, a clear shift after a stress of 438.2 MPa was measured, as shown in Figure 5.

By calculating the piezoresistive coefficient with Equation 2, it can be concluded that the piezoresistive coefficient of the RTD on the GaAs-on-Si substrate was in the range of 3.42 × 10^−9^ to 6.85 × 10^−9^ m^2^/N, which is about one order of magnitude higher than the Si-based semiconductor piezoresistors.

## Conclusions

In conclusion, we present a method to fabricate GaAs-based RTD on Si substrate. Due to high sensitivity to external stress, GaAs has a much higher piezoresistive coefficient than Si-based piezoresistors. Combining with RTD, the piezoresistive coefficient has reached more than one order of magnitude higher than Si. This work has combined the high strain sensitivity of GaAs-based RTD with the Si substrate. This will further provide us a possibility to develop some high-performance MEMS sensors.

## Competing interests

The authors declare that they have no competing interests.

## Authors’ contributions

JL (Jie Li) and HG fabricated the RTD-Si films, performed the measurements, and wrote the manuscript. JT and YS analyzed the results and wrote the manuscript. HN, CX, and ZN helped grow and measure the films. ML and YY helped measure the RTD-Si device. JL (Jun Liu) and WZ supervised the overall study. All authors read and approved the final manuscript.

## Authors’ information

JL (Jie Li) was born in 1976 in Shanxi, China. He received his Ph.D. in physics from the Beijing Institute of Technology, Beijing, China in 2005. He has published papers on topics including semiconductor materials, devices, and MEMS sensors. His current research interests include MEMS sensors and semiconductor physics. HG was born in 1987 in Shanxi, China. He is a graduate student at the School of Electronics and Computer Science and Technology, North University of China. His current research is focused on the field of semiconductor materials. JL (Jun Liu) was born in 1968 in the Inner Mongolia Autonomous Region, People's Republic of China. He received his Ph.D. degree from Beijing Institute of Technology, Beijing, China in 2001 and worked as a postdoctoral researcher in Peking University from 2003 to 2007. His research interests focus on MEMS and MIMU. As the team leader, he has worked on around 20 different projects funded by the National ‘863’ Project, National Nature Funds, National 973 Project, etc. He is now working as the director of The Ministry of Education Key Laboratory for Instrumentation Science & Dynamic Measurement at the North China Institute of Technology and the secretary general of Chinese Academy of Ordnance Industry. JT received his Ph.D. from the National Technical University of Athens. He is now working in the Key Laboratory of Instrumentation Science & Dynamic Measurement (North University of China), Ministry of Education. His current research interests include the self-assembly of nanomaterials, bio-chemical sensor applications, MEMS/NEMS, and inertial sensor design. YS was born in 1972 in Shanxi, China. He received his M.Sc. degree in electronic engineering from the North University of China, Shanxi, China in 2003. He has published papers on topics including microinertia device design and MEMS device design. His current research interests include microinertia navigation systems and MEMS sensors.
